# Nonequilibrium Magneto-Conductance as a Manifestation of Spin Filtering in Chiral Nanojunctions

**DOI:** 10.1021/acs.jpclett.3c01922

**Published:** 2023-08-30

**Authors:** M. A. García-Blázquez, W. Dednam, J. J. Palacios

**Affiliations:** † Departamento de Física de la Materia Condensada, Universidad Autónoma de Madrid, E-28049 Madrid, Spain; ‡ Department of Physics, Science Campus, University of South Africa, Florida Park, Johannesburg 1710, South Africa; ¶ Condensed Matter Physics Center (IFIMAC), Universidad Autónoma de Madrid, E-28049 Madrid, Spain

## Abstract

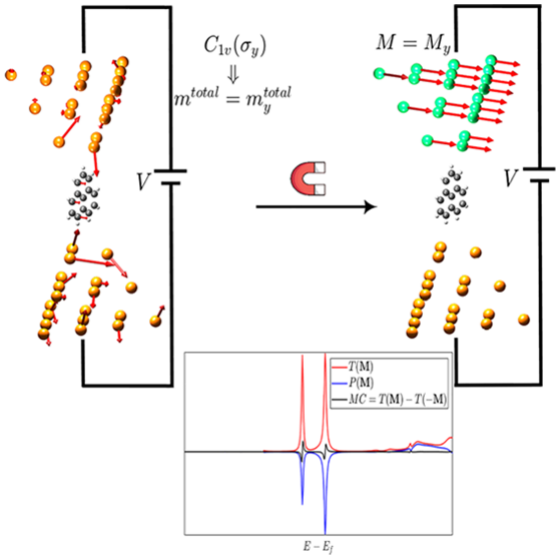

It is generally accepted that spin-dependent electron transmission may appear in chiral systems, even without magnetic components, as long as significant spin–orbit coupling is present in some of its elements. However, how this chirality-induced spin selectivity (CISS) manifests in experiments, where the system is taken out of equilibrium, is still debated. Aided by group theoretical considerations and nonequilibrium DFT-based quantum transport calculations, here we show that when spatial symmetries that forbid a finite spin polarization in equilibrium are broken, a *net* spin accumulation appears at finite bias in an arbitrary two-terminal nanojunction. Furthermore, when a suitably magnetized detector is introduced into the system, the net spin accumulation, in turn, translates into a finite magneto-conductance. The symmetry prerequisites are mostly analogous to those for the spin polarization at any bias with the vectorial nature given by the direction of magnetization, hence establishing an interconnection between these quantities.

Relativistic effects experienced by electrons propagating through matter, most importantly in the presence of heavy atoms, are essential for many of the intrinsic magnetic properties of a variety of systems. In particular, in combination with the breaking of inversion symmetry in bulk materials, spin–orbit coupling (SOC) translates into the appearance of spin textures in reciprocal space and enables nonequilibrium phenomena such as spin-charge conversion, spin accumulation, and magneto-conductance (MC).^1−4^ In solids, the spin Hall effect^5,6^ or the Edelstein effect,^7^ among others, results from it. More recently, chirality-induced spin selectivity (CISS),^8−13^ a seemingly related effect by which electrons propagating through chiral junctions (often involving chiral molecules) get spin polarized on average, has been proposed as an enabler for spintronics and quantum computing applications.^14−18^ The CISS effect is typically revealed as a finite MC in two-terminal devices by introducing a ferromagnetic detector.^19−21^

Over the years, a number of studies in the limit of zero bias have already established the crucial role of SOC in the emergence of a finite spin-polarization of the transmitted electrons in chiral junctions.^20,22−26^ The importance of the metallic contacts in molecular junctions stems then from the introduction of a sufficiently strong SOC but, notably, also from the modification of the symmetry of the system as a whole, thus enabling for a finite spin polarization along certain otherwise forbidden directions for the molecule or material alone.^27,28^ In this regard, rotational symmetries along the transport direction play the same role as mirror symmetries (whose absence is alluded to in the chirality term) depending on the specific direction of spin polarization.

On the other hand, in order to observe actual charge or spin currents, the system must be driven out of equilibrium. In our case, a bias voltage between the two terminals must be applied.^29−31^ Fundamentally, restrictions on the conductance in the unitary scattering scheme for coherent transport forbid the existence of a finite MC in two-terminal devices at equilibrium.^32,33^ This so-called Onsager relation does not hold, however, under out-of-equilibrium conditions (the magneto-current being nonlinear in the voltage^34,35^) where one would expect to observe the CISS effect, among other interesting spin phenomena. Nonetheless, self-consistent nonequilibrium calculations from density-functional theory (DFT) in this context have barely been explored so far,^36,37^ in contrast with their equilibrium counterparts.

In this work, we perform a series of SOC-corrected DFT nonequilibrium Green’s function (NEGF) quantum transport calculations with the aim of illustrating the interrelations among spin polarization, spin accumulation, and MC, as predicted by representation theory. First we perform the latter analysis within the NEGF formalism, determining the possible directions of spin polarization, accumulation, and MC (for which the vector nature is given by the ferromagnetic direction) according to the symmetries of the complete junction (reflections, accounting for the notion of chirality and rotations not permuting the electrodes). Since the selection rules are essentially coincident, the spin polarization, in or out of equilibrium, must be accompanied by the accumulation of a net spin density at finite bias, ephemeral though this may be,^29,37^ which can then interact with the magnetic elements yielding a finite MC. With these theoretical and computational results, we aim at elucidating the nonequilibrium spin-dependent response of nanoscopic systems in connection with equilibrium studies. Our quantitative results may nevertheless need to be complemented by inelastic effects such as electron–phonon interactions, which are currently deemed important to explain the large measured values and their scaling with temperature.^38−41^

The general framework of the nonequilibrium spin transport, on which the following analysis is based, is introduced in Computational Methods. The system is in general described by a spin-dependent Hamiltonian of the form *Ĥ*(***r***) = *Î*_*s*_ ⊗ *ĥ*^0^(*r̂*) + σ̂^*x*^ ⊗ *ĥ*^*x*^(***r***) + σ̂^*y*^ ⊗ *ĥ*^*y*^(***r***) + σ̂^*z*^ ⊗ *ĥ*^*z*^(***r***). Under any (active) transformation *g* of the point group  of the whole system, the Hamiltonain remains invariant:
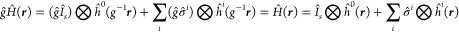
It follows immediately that *ĥ*^0^(*g*^–1^***r***) = *ĥ*^0^(***r***). Furthermore, noting that the components of the Pauli vector transform as components of an angular momentum, i.e.,  with  the weight-1 representation of *O*(3) (here restricted to ), which is even under inversion, it follows that  since  has real entries. In particular, this is the case for the spin–orbit interaction, *h*_SOC_^*i*^(***r***) ∝ (∇*V*(***r***) × ***p***)_*i*_, but it must hold irrespective of which spin-dependent terms are present in the Hamiltonian (albeit these may reduce  to a proper subgroup, unlike SOC). Let the retarded Green’s function *Ĝ*^+^(***r***) = *Î*_*s*_ ⊗ *ĝ*^0^(***r***) + ∑_*i*_σ̂^*i*^ ⊗ *ĝ*^*i*^(***r***), where the *E* variable has been omitted for brevity. Then, transforming by  in eq 6 and inspecting the scalar component (*Î*_*s*_ term)
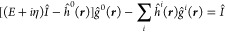
one concludes that *ĝ*^0^(*g*^–1^***r***) = *ĝ*^0^(***r***),  also.

In equilibrium, taking the spatial *i*th component in eq 9 and exploiting the invariance of the spatial integrals under orthogonal transformations , one obtains the conditions followed by the spin texture (expressed in terms of the overlap and one-particle density matrix, see eq 9)
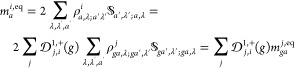
1where *ga* labels the device atom located at *g****t***_*a*_ (which must exist for all ), and we have used the unitarity of the representations of the basis functions φ_μ_ (which make them cancel in the λ, λ′ summations, irrespective of the specific orbital character) and the fact that ρ̂^eq,*i*^ transforms according to . We have also defined the device region as being invariant under , without loss of generality. Since  yields a bijection between the set of device atoms and itself, taking the sum over *a* ∈ *D* in eq 1 one obtains for the total spin density
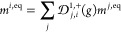
2For the mirror planes σ_*k*_,
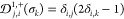
for any set of three orthogonal directions so that *m*^*i*^ = *m*^*i*^δ_*i*,*k*_. On the other hand, for a 2π/*n*–rotation *C*_*n*,*k*_ along the direction *k*,  coincides with the standard rotation matrix, and by applying eq 2 with *C*_*n*,*k*_ and *C*_*n,k*_^–1^, one concludes that *m*^*i*^ = *m*^*i*^δ_*i*,*k*_. We refer to these spatial symmetries that do not permute the electrodes as longitudinal. The total spin density must therefore point, as a (pseudo)vector quantity, perpendicular to any longitudinal plane of symmetry and along the axis of any nontrivial rotation symmetry. In particular, the simultaneous presence of a plane and an axis of symmetry forces ***m*** to vanish identically. Notice that this result also holds for each individual atomic layer in the junction (or, more precisely, for each subset of atoms that is invariant under the corresponding symmetry operation). Additionally, the spin density of each individual atom that is invariant under , i.e., *a* = *g*′*a*, is subject to the selection rule (eq 2) (for *g*′ only) of the total density.

The previous discussion in the equilibrium case, including eqs 1 and 2, is analogous for the out-of-equilibrium case with one exception: the symmetry operations must be limited to those that do not permute the electrodes (longitudinal). The operations that permute the electrodes (transversal) are actually not symmetries anymore due to the different normalization of the electronic density (in the DFT formalism) in both electrodes. The longitudinal symmetries form a subgroup of the original group without bias, namely,  where  is the point group of electrode *C* (sharing its invariant point with ). To prove the nonequilibrium case, we first note that the scalar and pseudovector transformation rules are preserved under multiplication, that is, *f̂*^0^(*g*^–1^***r***) = *f̂*^0^(***r***) and  with
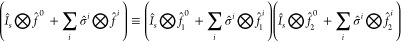
and *f̂*_1,2_^0^, *f̂*_1,2_^*i*^ transforming analogously. Then, by transforming in all matrix elements of eq 12 by , one obtains for the components in the *Î*_*s*_, σ̂^*i*^ decomposition of spin space; Σ̂_*C*_^0^(*g*^–1^***r***) = Σ̂_*C*_^0^(***r***),
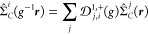
with *C* = *A*, *B*. By performing these operations in eq 14, one then concludes that the spin components of *Ĝ*^<^ have the same transformation properties under longitudinal operations as those of *Ĝ*^+^. For transversal symmetries without bias, employing eqs 10 and 13 and proceeding as above, one obtains a generalized transformation rule reversing the bias voltage. Combining it with that for longitudinal symmetries,
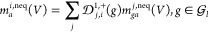
3
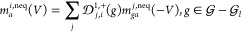
4

Therefore, the restrictions induced by longitudinal symmetries (eq 3) on the total spin density out of equilibrium are ultimately equal to those on the spin polarization, which can be derived with the unitary scattering formalism in the linear regime^27^ and extended to the nonlinear regime, noting that Green’s functions in the Caroli formula (eq 5) transform equally under longitudinal operations.

On the other hand, under time-reversal symmetry Θ̂*Ĥ*(***r***)Θ̂^–1^ = *Ĥ*(***r***), where Θ̂ = (σ̂^*y*^ ⊗ *Î*)*K̂* up to an arbitrary phase and *K̂* acts as complex conjugation. Then, performing this antiunitary transformation in the expression for a general Green’s function of complex argument *z*,
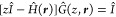
it follows that Θ̂*Ĝ*(*z*, ***r***)Θ̂^–1^ = *Ĝ*(z*, ***r***) = *Ĝ*^†^(*z*, ***r***). In particular, Θ̂*Ĝ*^+^(*E*, ***r***)Θ̂^–1^ = *Ĝ*^–^(*E*, ***r***), the advanced Green’s function. Thus, for the equilibrium density matrix (eq 10),
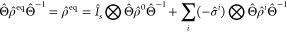
Then Θ̂ρ̂^0^Θ̂^–1^ = ρ̂^0^, Θ̂ρ̂^*i*^Θ̂^–1^ = −ρ̂^*i*^, and by transforming in the scalar products of eq 9, one concludes that ***m***_*a*_ = 0 for all atoms and obviously ***m*** = 0. In the absence of magnetic elements (and fields) in the junction, the spin density at equilibrium is therefore locally vanishing. For the magnetization (eq 9) in the nonlinear regime, from eqs 12, 14, and the above, it follows that
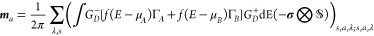
and an analogous result holds for the total spin density (eq 8). In contrast to the linear regime, this generally does not result in an actual restriction of the spin density.

Note that both time reversal and the spatial operations are here formally defined as symmetries according to the single-particle Hamiltonian of the system; i.e., they are intrinsic to the system irrespective of the applied bias, which then modifies the effect of the operations that change the propagation (Θ and the transversal symmetries) in the NEGF framework.

We have performed a series of DFT-based nonequilibrium quantum transport calculations to compute the spin density and polarization in this regime (computational details can be found in the Computational Methods section). Two illustrative cases are considered here: bare W nanocontacts (see Figure 1a–1b) and a molecular bridge composed of Pb electrodes and a triangulene molecule (see Figure 1d–1e). The scattering regions in the calculations exactly include the atoms depicted in the corresponding figures.

**Figure 1:**
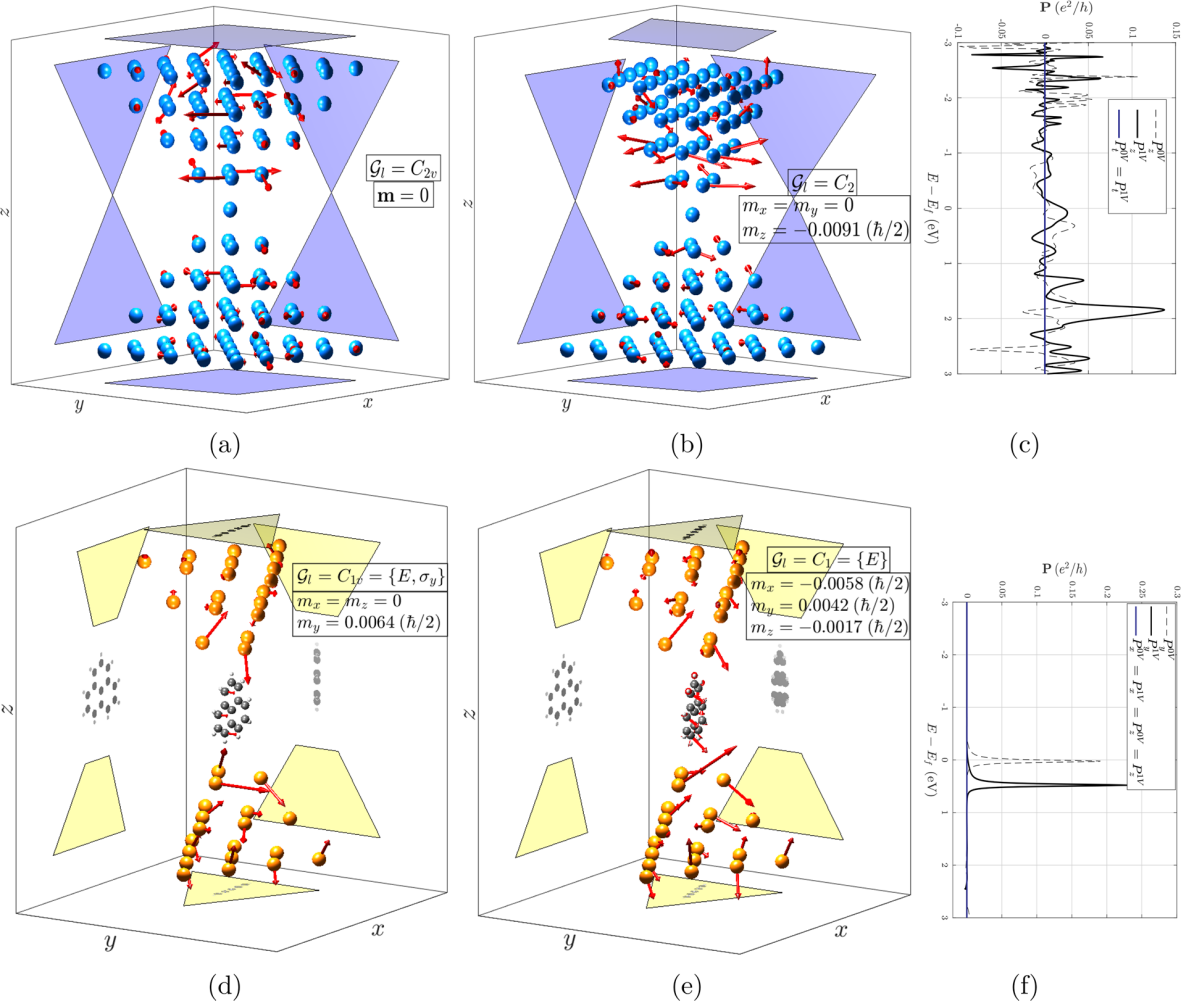
Nonequilibrium spin density per atom with bias 1 V. *x*, *y*, and *z* projections are shown to help visualize the structures, with the top and bottom contacts having a separate projection above and below (respectively) the structure. The magnitude of the vectors is purely illustrative and has been uniformly scaled by the same factor in (a,b) and (d,e) to make them clearly visible. (a) Bare W contacts aligned, point group , group of longitudinal symmetries . (b) Bare W contacts misaligned, . (d) Pb contacts with an aligned triangulene molecule, . (e) Pb contacts with a misaligned triangulene molecule, . (c,f) Equilibrium (0 V) and nonequilibrium (1 V) spin polarization components of, respectively, the systems in (b,d), where *P*_*t*_ stands for any transversal direction (⊥ ***z***). SOC is included only in the contacts, excluding the shown layers of maximum and minimum *z*.

As can be observed, a finite spin density is accumulated per atom in the presence of a bias voltage (except if such an atom is invariant under both a longitudinal plane σ_*l*_ and axis *C*_*n*,*l*_, as are, e.g., all atoms in a monatomic chain). In these nonmagnetic systems, the spin density is a purely nonequilibrium quantity induced by the SOC. The net density is, however, strictly vanishing for aligned crystallographic electrodes (at least for those that are cubic, tetragonal, or orthorhombic in the bulk), such as in Figure 1a, due to the presence of both symmetries  as dictated by eq 3. Upon a relative rotation of a single contact, the mirror plane is generally removed and a net magnetization along the transport direction is hence enabled; see Figure 1b.

Likewise, in the molecular bridge of Figure 1d only σ_*l*_ or, in the chosen coordinate system, σ_*y*_ is a symmetry. Thus, by eq 3 the net spin density must point perpendicular to the plane: ***m*** = *m*_*y*_***y*^**. For each atom that lies in such a plane, in particular, the whole molecule, the same rule applies individually. Note that the asymmetry of the electrodes is inherited in the spin accumulation of the molecule, which is by itself achiral with, in the present orientation with respect to the longitudinal direction,  and would thus present a vanishing ***m*** if the geometry of the electrodes was ignored. Numerically, |***m***_*a*_| in the molecule can be comparable to or larger than that in other heavier atoms of the electrodes even if the SOC strength of the former is negligible in comparison. By analogy to Figure 1b, the rotation of the molecule with respect to the contacts will generally break the longitudinal mirror symmetry, allowing for a finite net spin density in arbitrary directions; see Figure 1e (note that in this case there are no spatial symmetries at all).

In Figure 1c–1f, the polarization both in and out of equilibrium for the systems in Figure 1b and 1d is depicted. As can be observed, the numerical behavior of this quantity with the bias voltage is quite specific to the given system, but as expected, the symmetry rules enforcing the vanishing of any ***P*** components do remain valid in the nonlinear regime. Note that the finite components of net spin density and polarization, the latter either in or out of equilibrium, are indeed the same.

The total transmission (or dimensionless conductance) in a system with a ferromagnetic component of macroscopic magnetization ***M***, namely, *T*_***M***_ = *T*_***M***_^↑↑^ + *T*_***M***_^↑↓^ + *T*_***M***_^↓↑^ + *T*_***M***_^↓↓^, can be computed from the spin-resolved Caroli formula at a given energy:
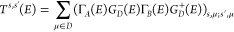
5This expression is valid for both in- and out-of-equilibrium situations, with differences in Green’s functions induced from the self-consistent Hamiltonian through the charge density. The difference in conductance for opposite magnetizations, *ΔT*_***M***_ = *T*(***M***) – *T*(−***M***), is the so-called magneto-conductance (MC). This quantity, when measured in experiments with chiral and ferromagnetic components, is often considered a manifestation of the CISS effect.^19−21^ Note that this differs from the tunnel magneto-conductance, for which the two magnetic configurations are not reversed as a whole. At equilibrium, a finite MC in two-terminal devices is forbidden by Onsager’s reciprocity, which can be proved within the scattering formalism for coherent transport by performing the time-reversal operation (not a symmetry for finite ***M***) and invoking the unitarity of the scattering matrix.^33^ This formalism no longer holds in the nonlinear regime, allowing in principle for a finite MC if spatial symmetries do not forbid it.

Consider a junction with a ferromagnetic component magnetized along the longitudinal direction, say ***M*** = *M****z***, which presents a longitudinal plane of symmetry σ^*l*^ if magnetism is disregarded (or equivalently, Θ*σ*^*l*^ is an element of the magnetic point group but not σ^*l*^). Then the Hamiltonian operator of the system must transform as *Ĥ*_***M***_(σ^*l*^***r***) = *Ĥ*_–***M***_(***r***) so that by applying this operation in the space integrals of eq 5 one obtains *T*_***M***_^*s*,*s*′^(*E*) = *T*_–***M***_^*s*, *s* ′^(*E*) with *s* being the opposite spin state of *s*.^27^ Therefore, in this case, *ΔT*_***M***_(*E*) = 0 even out of equilibrium. Similarly, when the magnetization ***M*** is along an arbitrary transversal direction, i.e., perpendicular to the transport direction, a longitudinal 2-fold rotation symmetry *C*_2,*z*_ (when disregarding magnetism) has the same effect as σ_*l*_ above. That is, the MC for transversal ferromagnets must vanish if Θ*C*_2,*z*_ is an (antiunitary) symmetry of the system, even in the nonlinear regime. Note that this argument does not hold for other rotations *C*_*n*,*z*_, *n* ≥ 3 (Θ*C*_*n*,*z*_ is not an antiunitary symmetry) since then the results for *C*_*n*,*z*_ and *C*_*n,z*_^–1^ yield different ***M*** vectors and cannot be combined.

Importantly, these selection rules for the MC are analogous to those for both the total spin density and the spin polarization (see the next paragraph) as discussed above, with the direction of the magnetization ***M*** playing the same role as the spatial component of ***m*** or ***P***. This is consistent with the expectation of detecting the net spin density along a given direction by introducing a ferromagnet oriented along that direction. As explained above, the only exception to these shared selection rules occurs for transversal directions in systems whose point group has a main (longitudinal) rotation axis of odd order; e.g.,  forces the vanishing of *P*_*t*_ and *m*_*t*_ along any transversal direction but not of *ΔT*_*M*_*t*__ (see the numerical example in the Supporting Information).

It may be worth noting that the introduction of the ferromagnet induces a finite spin polarization ***P***(***M***) regardless of whether the underlying ***P***(0), which is induced by SOC if spatial symmetries allow for it, is vanishing or not. The quantity that actually shares the selection rules with *ΔT*_***M***_, ***m*** and ***P***(0) (except for odd-order rotations), is the symmetrized Δ***P***_***M***_ = ***P***(***M***) + ***P***(−***M***).

**Table 1:** Selection Rules Summary^a^

Symmetry	***m***_*a*_^eq^	***m***^eq^	***m***_*a*_^neq^	***m***^neq^	*ΔT*_***M***_
Θ	0	0			Not a symmetry
σ	⊥σ: ***m***_*a*_^eq^ = ***m***_*σa*_^eq^	⊥σ	⊥σ_*l*_: ***m***_*a*_^neq^ = ***m***_σ_*l*_*a*_^neq^	⊥σ_*l*_	*ΔT*_***M***⊥σ_*l*__
	∥σ: ***m***_*a*_^eq^ = −***m***_*σa*_^eq^		∥σ_l_: ***m***_*a*_^neq^ = −***m***_σ_l_*a*_^neq^		(Θ*σ*_*l*_ symmetry)
			only for σ_*l*_		
*C*_*n*_	(1)	∥*C*_*n*_	(3) only for *C*_*n*,*l*_	∥*C*_*n*,*l*_	*ΔT*_***M***∥*C*_*n,l*__ , only for *n* = 2
					(Θ*C*_2,*l*_ symmetry)

a Subscript *l* means longitudinal, i.e., not permuting the electrodes.

The previous DFT calculations out of equilibrium can be expanded to compute the MC by introducing a ferromagnetic component. (Computational details can be found in the Computational Methods section.) Here we consider the Pb-triangulene molecular junction of Figure 1d–1e with a ferromagnetic Ni drain contact that does not change the original point groups when disregarding magnetism. Other illustrative systems may be found in the Supporting Information. The results are displayed in Figure 2, where the different ***M*** orientations and rotations of the molecule allow testing of the previous selection rules for the MC, in particular, for the longitudinal mirror symmetry (which chiral systems lack). Note that in our case the MC is a spin-related signal stemming purely from the SOC since in its absence (also of further spin-dependent terms other than the ferromagnetism) it would be *T*^↑↑^(±***M***) = *T*^↓↓^(∓***M***), *T*^↑↓^(±***M***) = *T*^↓↑^(∓***M***) = 0 so that *ΔT*_***M***_ = 0.

**Figure 2:**
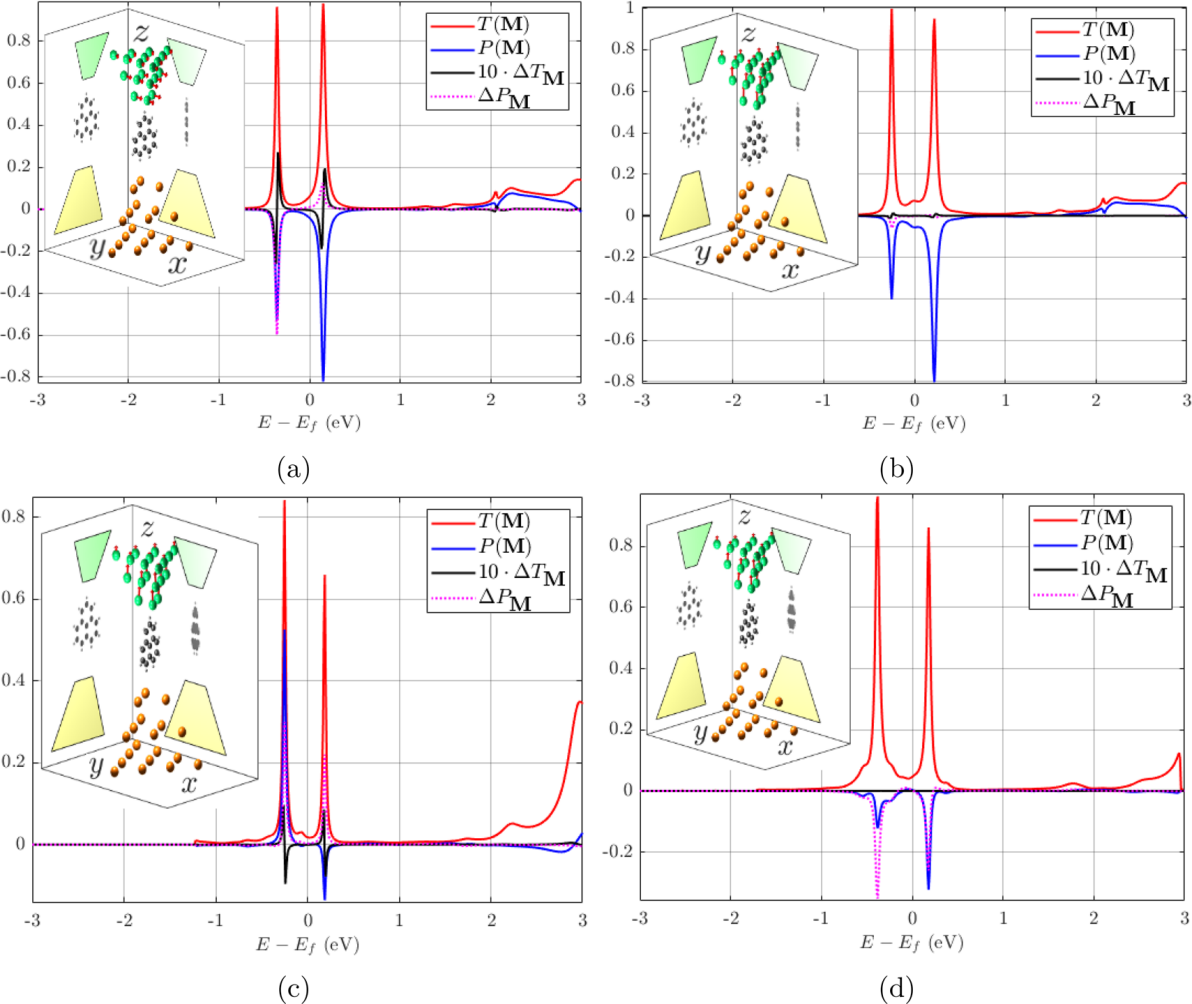
Dimensionless magneto-conductance (*ΔT*_***M***_ = *T*(***M***) – *T*(−***M***)) and symmetrized spin polarization (Δ***P***_***M***_ = ***P***(***M***) + ***P***(−***M***), component ***P***∥***M***) in junctions with Pb source contact with *C*_3*v*_ symmetry, a triangulene molecule, and a ferromagnetic (***M***) Ni drain contact with *C*_3*v*_ (disregarding magnetism) in different orientations of both the molecule and ***M***. Insets show the system in each subfigure, including the (ferro-)magnetization per Ni atom in red arrows whose length is illustrative. (a) Molecule aligned with the contacts, ***M*** = *M*_*y*_***y*^**, point group  bias 1*V*. (b) Same as (a) except for ***M*** = *M*_*z*_***ẑ*** and point group . (c) Same as (b), with molecule rotated by 15°, trivial point group . (d) Same as (c) except for no bias (0 *V*). SOC is included only in the contacts, excluding the shown layers of maximum and minimum *z*. In all cases, both the SOC strength in Ni and the values of the MC have been enlarged by a factor of 10 for clarity sake in the qualitative behavior of the spin signals. No symmetries have been explicitly used in the self-consistent calculations, hence the (comparatively) small but finite *ΔT*_***M***_, Δ***P***_***M***_ in (b).

It can be observed in Figure 2a that the MC is indeed finite for ***M*** perpendicular to the symmetry plane σ_*y*_, i.e., in a transversal direction. On the other hand, the MC is remarkably smaller (theoretically vanishing) for ***M*** along the transport direction (Figure 2b) since the latter is contained in σ_*y*_. The fact that MC and Δ***P***_***M***_ are not strictly vanishing in this case should be explained by the fact that the mirror symmetry is not explicitly used in our self-consistent calculations. Upon rotation of the molecule, the point group of the junction can be made trivial, thus enabling the emergence of MC along the (previously forbidden) transport direction; see Figure 2c. Onsager’s relation is verified in Figure 2d for the latter asymmetric system showing that MC cannot appear in equilibrium, even in the presence of a finite spin polarization without magnetism. As observed, this result is numerically robust against the absence of symmetries; see further examples in the Supporting Information. Recalling Figure 1d–1e, the MC indeed coexists with a net spin density out of equilibrium. Furthermore, these two quantities, in turn, coexist with a finite Δ***P***_***M***_ and the underlying ***P***(***M*** = 0), which represents the smoking gun without magnetism and with or without applied voltage.

The relative size of the MC is most certainly small (∼1–2% in Figure 2a, 2c) considering the enlarged SOC factor (×10) in the Ni electrode. Previous studies with tight-binding models for helicene molecules have reported values on the order of 0.1%,^35,42^ which are consistent with our results in a similar system employing the original SOC strength (see the Supporting Information). The inclusion of many-body corrections is thought to yield significantly higher values, in particular, for the on-site Hubbard interaction in helicene atoms,^43^ and is positively correlated with the length of the molecule. We believe that an analogous increase could potentially be achieved by enlarging the electrodes, thereby increasing the number of atoms with a strong SOC and possibly the contact area with the ferromagnetic component. Studies to quantify the extensive nature of the MC with respect to all of the components of the junction could, therefore, shed further light on the actual size of the CISS effect in general systems.

In summary, we derive a complete set of symmetry restrictions on both spin density and MC that are valid out of equilibrium and confirm and illustrate them via magnetic DFT quantum transport calculations, with a self-consistent treatment of the bias voltage. The numerical results for helical molecules are consistent with the literature. These selection rules emphasize and determine the important role of the geometry of the system as a whole and help identify the underlying relations between the central quantities in spin transport phenomena, such as the CISS effect. In particular, the presence of a finite spin polarization in (or out of) equilibrium along a given direction without magnetic elements indicates that two other finite quantities will arise out of equilibrium: (1) a net spin accumulation in the system along the same direction and (2) an MC upon introduction of a ferromagnetic detector with magnetization along the same direction. These two quantities may then be regarded as an out-of-equilibrium manifestation of the spin polarization in the linear regime.

## Computational Methods

First we discuss the framework of nonequilibrium spin transport, on which the main text is based. Consider a junction with open boundary conditions described by a spin-dependent Hamiltonian operator *Ĥ*(***r***) and its corresponding retarded Green’s function
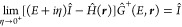
6where *Î* is the identity operator. Extensive quantities for the system may be computed from the one-particle density operator ρ̂ as ⟨*Â*⟩ = Tr[ρ̂*Â*]. Consider a finite, atom-centered set of basis functions φ_μ_(***r***) = φ_*a*,λ_(***r***) = φ_λ_(***r*** – ***t***_*a*_) ≡ ⟨***r***||*a*, λ⟩ = ⟨***r***||μ⟩ with overlap matrix , where ***t***_*a*_ is the coordinate vector of atom *a* and μ = (*a*, λ) is a general multi-index for the usual basis parameters (*a* → atoms, λ = λ(*a*) → shells and functions therein). The basis is doubled to account for the spin degree of freedom *s*∈{↑, ↓}: φ_μ_^*s*^(***r***) = φ_μ_(***r***)|*s*⟩ ≡ ⟨***r***||*s*, μ⟩, yielding the block diagonal overlap matrix . Upon restriction to a spatial subsystem *D*, in particular, the device or scattering region of the junction, a non-Hermitian projection^44^ is taken for consistency with the Mulliken population analysis^45^ (equivalent to setting *Â* = *Î*):
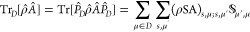
7Here the projector has been defined as  and ρ, *A* are the matrix representations of ρ̂, *Â* in the dual basis.^44^ (Throughout the text, summations of basis indices in an unspecified range (such as ∑_μ_) are meant to span the whole basis.)

Setting ***Â*** = **σ̂** ⊗ *Î* in eq 7, with **σ** = (σ^*x*^, σ^*y*^, σ^*z*^) being the Pauli vector, one then obtains the magnetization or spin density
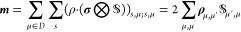
8where in the last step we have expanded ρ̂(***r***) = *Î*_*s*_ ⊗ ρ̂^0^(***r***) + ∑_*i*_ σ̂^*i*^ ⊗ ρ̂^*i*^(***r***) in the two-dimensional spin space, with *Î*_*s*_ being the identity there, and **ρ̂** = (ρ̂^*x*^, ρ̂^*y*^, ρ̂^*z*^). (For simplicity, we employ the notation *Î* for the identity operator both including and excluding the spin space. *Î*_*s*_ denotes the identity on the spin space alone. We have also omitted the global factor ℏ/2 for brevity.) This corresponds to the total spin density in the device, which is the sum of the contributions of all atoms in that region. Each individual atomic spin density is thus evaluated as
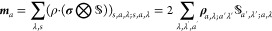
9At equilibrium (linear regime), the leads present the same electrochemical potential μ and the density operator ρ̂^eq^ can be related to the retarded and advanced [*Ĝ*^–^ = (*Ĝ*^+^)^†^] Green’s functions as
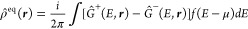
10where *f* is the Fermi distribution function. As usual, the retarded Green’s function matrix block (dual basis) in the device can be expressed in terms of the device Hamiltonian and the lead self-energies Σ_*A*_, Σ_*B*_ as
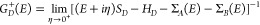
11where
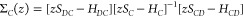
12for *C* = *A*, *B*.

Upon application of a bias *V* to the junction, a difference in electrochemical potentials μ_*B*_ – μ_*A*_ = *eV* is established between the electrodes *A* and *B*. The Fermi function in eq 10 is then updated to *f*(*E* – min(μ_*A*_, μ_*B*_)), and the corresponding density matrix for the out -of-equilibrium (nonlinear) regime is ρ̂(***r***) = ρ̂^eq^ (***r***) + ρ̂^neq^(***r***), with^46,47^
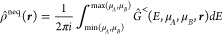
13where *Ĝ*^<^ is the lesser Green’s function, whose matrix representation (dual basis) in the device is
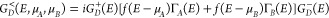
14with Γ_*C*_ = *i*(Σ_*C*_ – Σ_*C*_^†^) .

Note that eq 8 for the spin density is similar to that of the spin polarization,^48^ the latter employing the scattering density matrix describing the set of outgoing channels instead of the eigenfunctions in the device region.

Finally, we detail the most relevant aspects of the numerical calculations. The DFT-based quantum transport calculations have been performed with our code ANT,^47,49−51^ which is fully integrated in *Gaussian 09*.^52^ In ANT, the electrodes outside the scattering region (not depicted in the figures) are described by a tight-binding model on a Bethe lattice, which facilitates the calculation of self-energies while keeping the symmetry of the system intact.^47^ As described in references (53) and (54), SOC is included as a postselfconsistency first-order perturbation correction with prior optimization^55^ of typically large Gaussian basis sets^56−59^ for the purpose of faithfully reproducing the electronic structure of the bulk electrodes. The usual Perdew–Burke–Ernzerhof (PBE) exchange-correlation functional^60^ is used on account of its relatively low computational cost and ability to reproduce the electronic structure of metals reasonably well. The underestimation of the molecular gaps is of no concern here.

The equilibrium and out-of-equilibrium spin densities are obtained following eqs 8–14 employing a self-consistent Kohn–Sham Hamiltonian computed with *Gaussian 09*,^52^ imposing a convergence tolerance of 10^–7^ Ha. In the absence of SOC, the calculation of the equilibrium part of the spin density can be efficiently carried out by taking the integration contour along a semicircumference in the upper complex half-plane (similar to the evaluation of the charge density in the self-consistent procedure). However, upon the addition of SOC the Hamiltonian is no longer real and symmetric, hence the retarded and advanced Green’s functions must be integrated separately in the upper and lower complex half-planes, respectively, as explained in reference (61). Details of the nonequilibrium implementation prior to the addition of SOC, where the integration in the bias window needs to be done along the real axis, is provided in reference (51), and this is unaltered by the addition of SOC.

In the MC calculations, the reversal of magnetization ***M*** is achieved by swapping the spin-diagonal blocks of the fully converged unrestricted Kohn–Sham Hamiltonian, prior to the addition of SOC, once the desired degree of self-consistency has been achieved. For simplicity’s sake and computational efficiency, we have considered here a minimal *sd* basis in all Ni atoms. As stated in the main text, the SOC strength in Ni has been consistently increased by a factor of 10 in Figure 2 in order to make the MC clearly visible alongside conductance and spin polarization in a single axis system. No explicit use of symmetries has been made in any MC calculations, both in the main document and in the Supporting Information.

## Supporting Information Available

Same quantities as in any subfigure of Figure 2 (dimensionless conductance, spin polarization, magneto-conductance, and sum of polarizations for opposite magnetizations) for the following systems: bare Ni–Ni contacts with different distortions or symmetries and spin directions (augmented SOC), bare W–W–Ni contacts with 3-fold rotation symmetry (original SOC), and molecular bridge with Pt and Ni contacts and a helical molecule (original SOC); for the latter system, a plot of the magneto-conductance as a percentage of the sum of conductances is also included (PDF)
Geometry files (ZIP)
Transparent Peer Review report available (PDF)
