# Supplementary material for: Nonequilibrium Magneto-Conductance as a Manifestation of Spin Filtering in Chiral Nanojunctions
Source: J Phys Chem Lett. 2023 Aug 30;14(35):7931–9. doi: 10.1021/acs.jpclett.3c01922 (PMC10494227; doi:10.1021/acs.jpclett.3c01922)
Supplement: Supplementary file 1 — jz3c01922_si_001.pdf [file jz3c01922_si_001.pdf]

Non-Equilibrium Magneto-Conductance as a Manifestation of Spin Filtering in Chiral Nanojunctions —SI

M. A. García-Blázquez,*,† W. Dednam,‡ and J. J. Palacios*,†,¶

† *Departamento de Física de la Materia Condensada, Universidad Autónoma de Madrid, E-28049 Madrid, Spain*

‡*Department of Physics, Science Campus, University of South Africa, Florida Park, Johannesburg 1710, South Africa*

¶*Condensed Matter Physics Center (IFIMAC), Universidad Autónoma de Madrid, E-28049 Madrid, Spain*

E-mail: manuelantonio.garcia@estudiante.uam.es; juanjose.palacios@uam.es

Further MC results

Ni(C_4)-Ni(C_4)

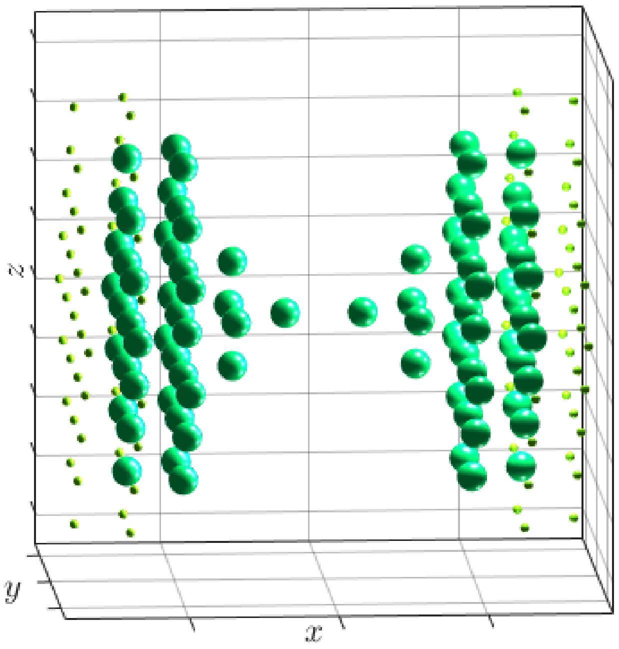

Figure 1: Reference structure with point group $\mathcal{G} = D_{4h}$ $(4/mmm)$

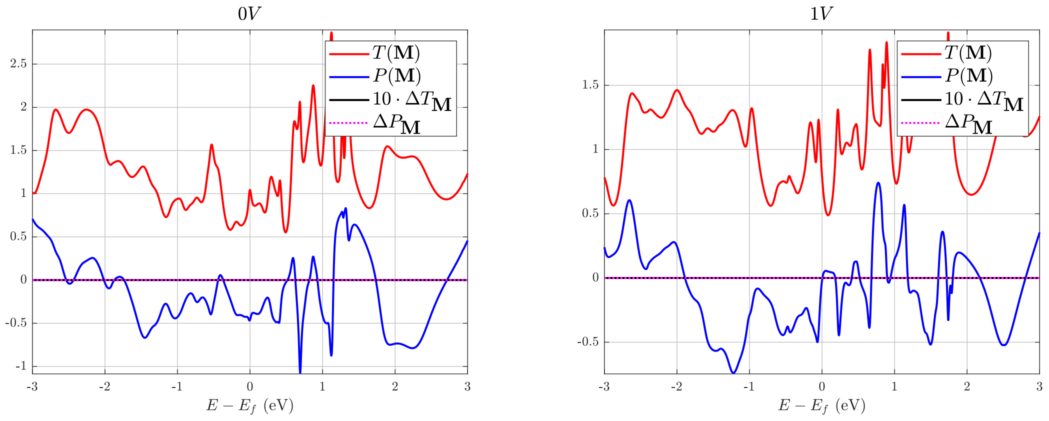

Figure 2: $\boldsymbol{M} \parallel \hat{\boldsymbol{z}}$ (transversal). Full symmetry: $\mathcal{G} = \begin{Bmatrix} D_{4h}, \text{ without magnetism} \\ m'm'm, \text{ with magnetism} \end{Bmatrix}$. Ni SOC $\times 10$

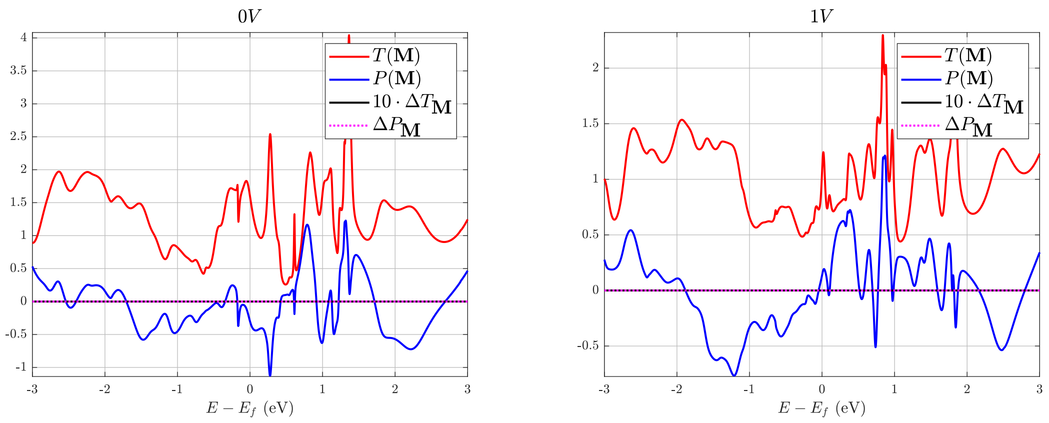

Figure 3: $\boldsymbol{M} \parallel \hat{\boldsymbol{x}}$ (longitudinal). Full symm.: $\mathcal{G} = \begin{cases} D_{4h}, \text{ w/o mag.} \\ mm'm', \text{ with mag.} \end{cases}$. Ni SOC $\times 10$

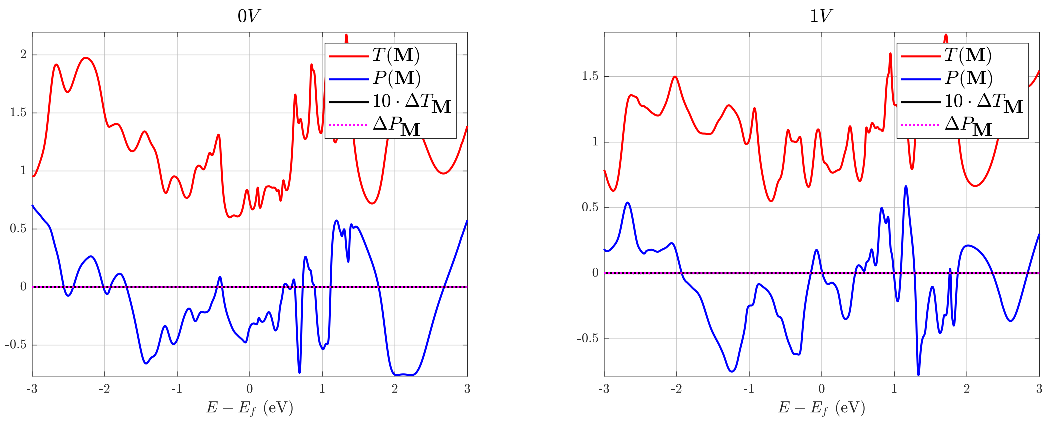

Figure 4: $\boldsymbol{M} \parallel \hat{\boldsymbol{z}}$ (transv.). Distorted: $\mathcal{G} = \begin{cases} C_{1v} = \{ E, \sigma_y \}, \text{ w/o mag.} \\ m' = \{ E, \Theta\sigma_y \}, \text{ with mag.} \end{cases}$. Ni SOC $\times 10$

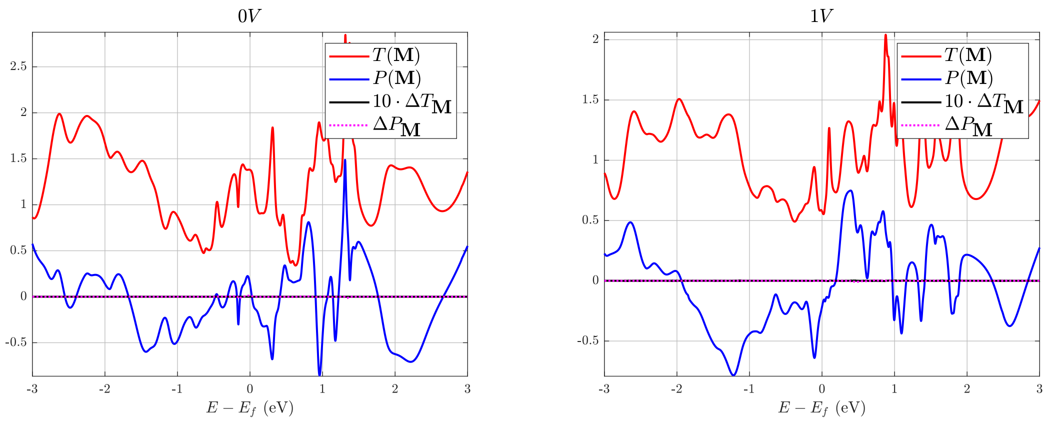

Figure 5: $\boldsymbol{M} \parallel \hat{\boldsymbol{x}}$ (long.). Distorted: $\mathcal{G} = \begin{cases} C_{1v} = \{ E, \sigma_y \}, \text{ w/o mag.} \\ m' = \{ E, \Theta\sigma_y \}, \text{ with mag.} \end{cases}$. Ni SOC $\times 10$

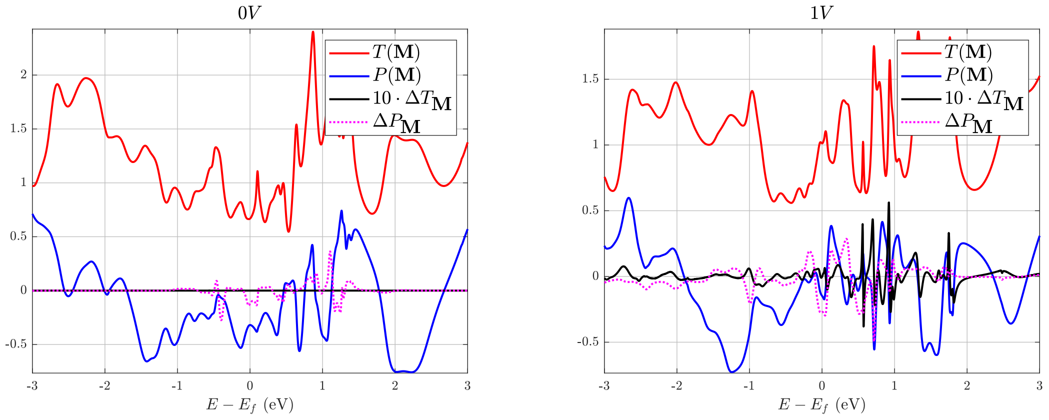

Figure 6: $\boldsymbol{M} \parallel \hat{\boldsymbol{y}}$ (transv.). Distorted: $\mathcal{G} = \{\, E, \sigma_y \,\}$. Ni SOC $\times 10$

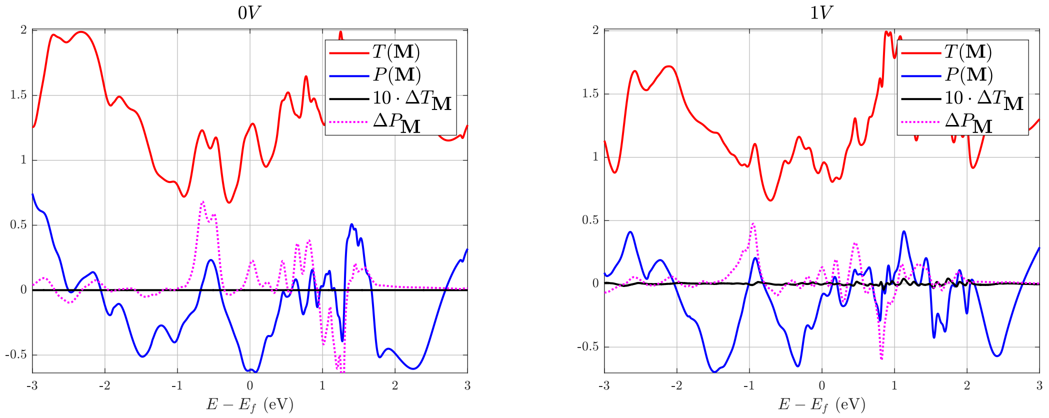

Figure 7: $\boldsymbol{M} \parallel \hat{\boldsymbol{z}}$ (transv.). Distorted: No symmetries. Ni SOC $\times 10$

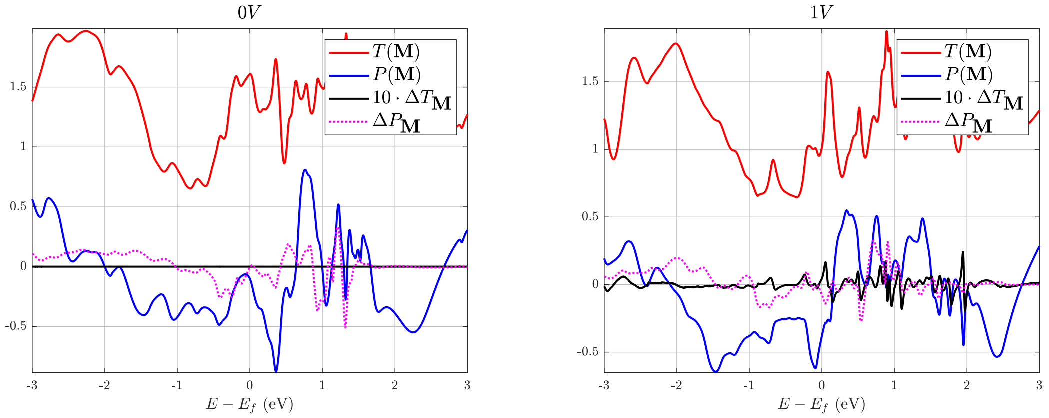

Figure 8: $\boldsymbol{M} \parallel \hat{\boldsymbol{x}}$ (long.). Distorted: No symmetries. Ni SOC $\times 10$

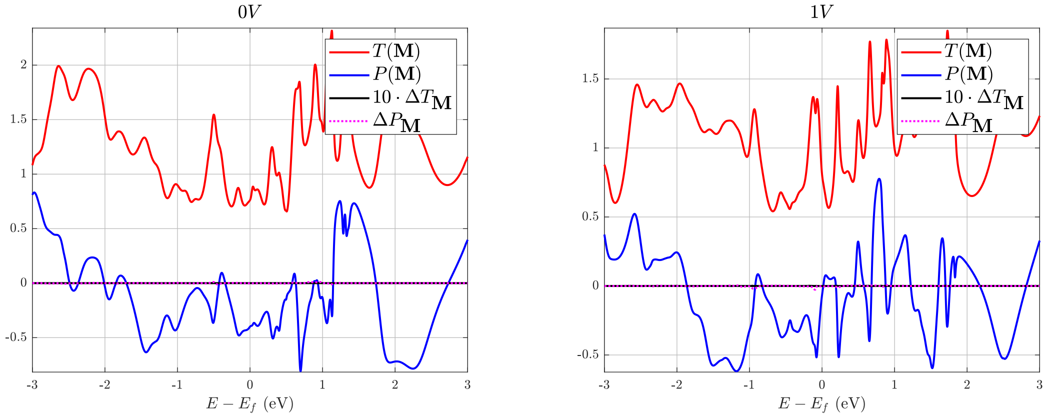

Figure 9: $\boldsymbol{M} \parallel \hat{\boldsymbol{z}}$ (transv.). Relative rotation of 15°: $\mathcal{G} = \left\{ \begin{array}{r} C_4 = \{\, E, C_{4x}, C_{2x}, C_{4x}^{-1} \,\}, \text{ w/o mag.} \\ 2' = \{\, E, \Theta C_{2x} \,\}, \text{ with mag.} \end{array} \right\}$. Ni SOC $\times 10$

W(C_3)-W(C_3)-Ni(C_3) (30° relative rotation)

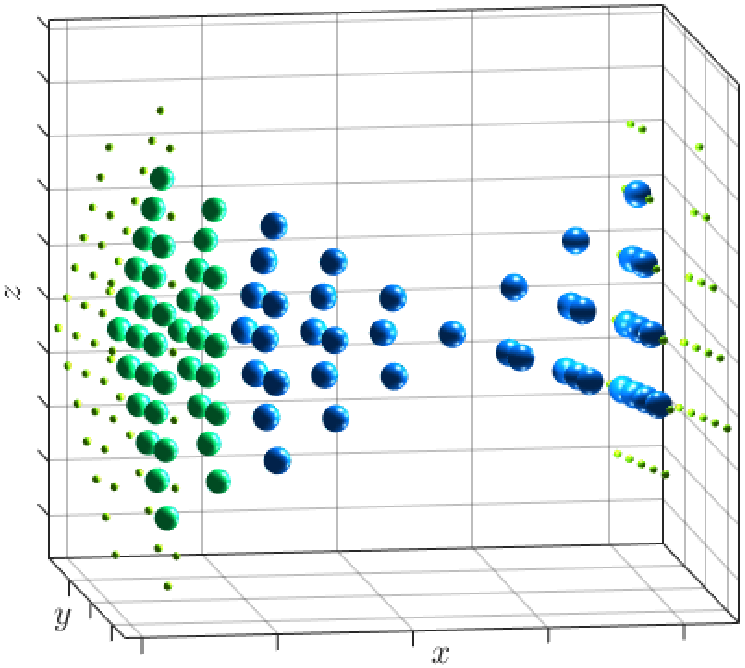

Figure 10

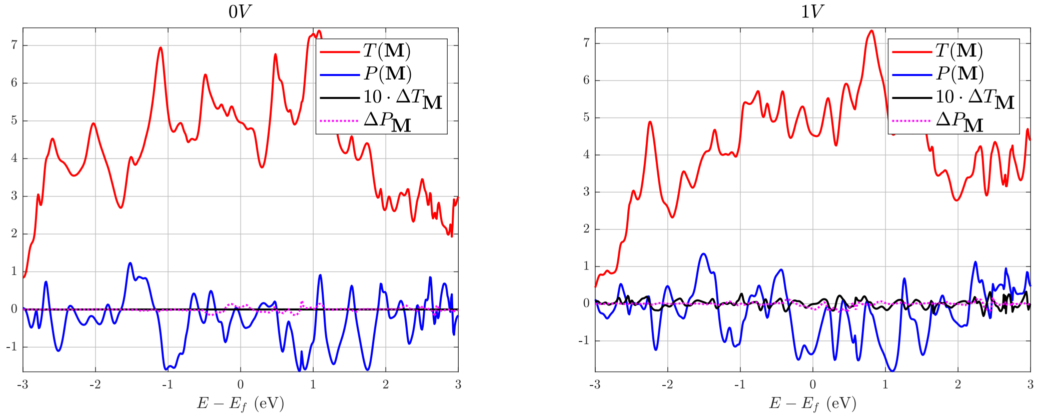

Figure 11: $\boldsymbol{M} \parallel \hat{\boldsymbol{z}}$ (transversal). $\mathcal{G} = \begin{Bmatrix} C_3 = \{\, E, C_{3x}, C_{3x}^{-1} \,\}\text{, without mag.} \\ \text{Trivial, with mag.} \end{Bmatrix}$. Unaltered SOC

Pt(C_4)-Helicene-Pt(C_4)-Ni(C_4)

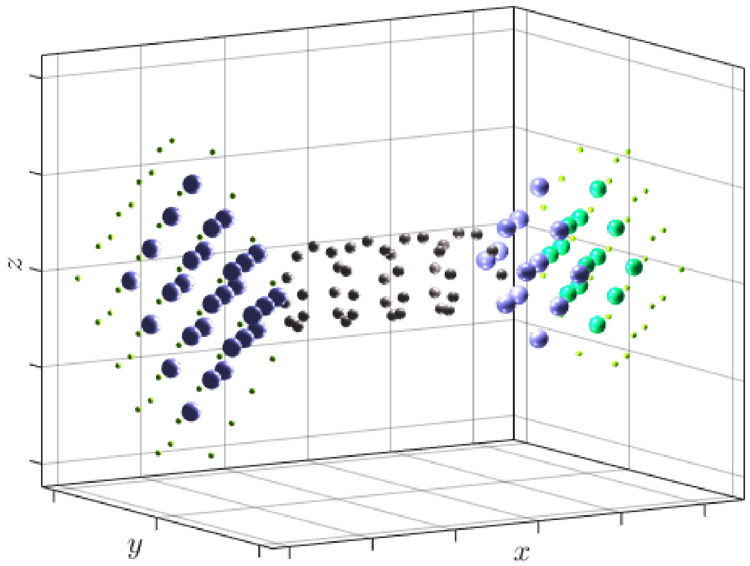

Figure 12

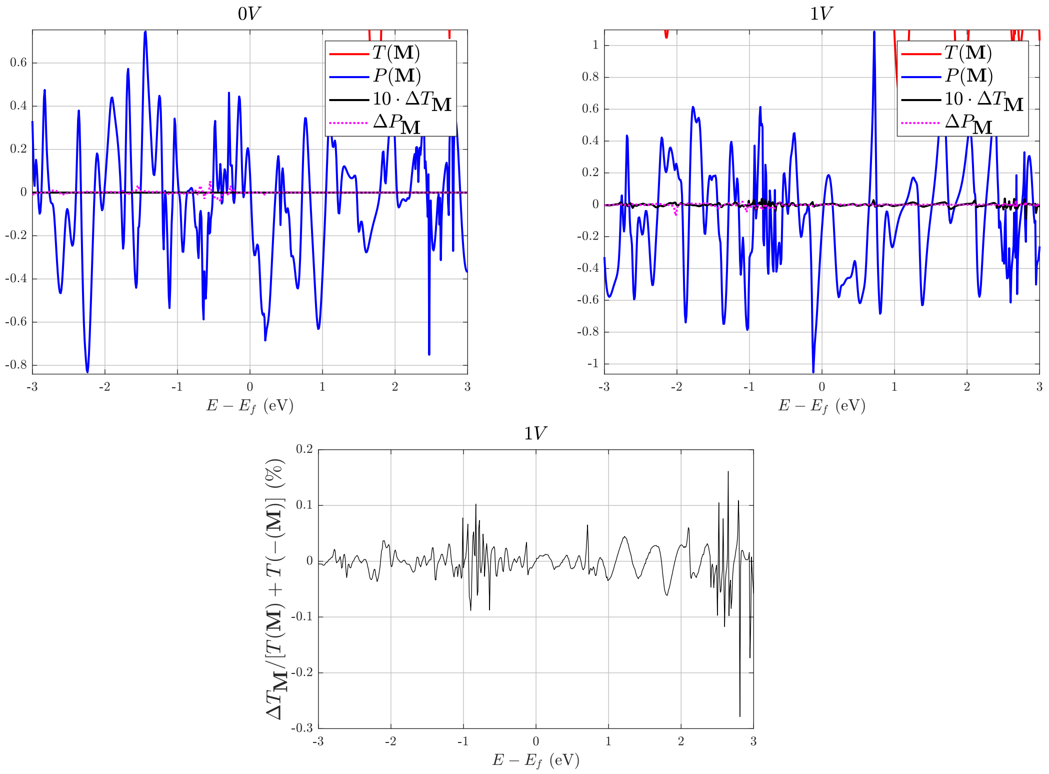

Figure 13: $\boldsymbol{M} \parallel \hat{\boldsymbol{z}}$ (transversal). No symmetries. Unaltered SOC
