# Supplementary material for: Nonequilibrium Magneto-Conductance as a Manifestation of Spin Filtering in Chiral Nanojunctions
Source: J Phys Chem Lett. 2023 Aug 30;14(35):7931–9. doi: 10.1021/acs.jpclett.3c01922 (PMC10494227; doi:10.1021/acs.jpclett.3c01922)
Supplement: Supplementary file 3 — jz3c01922_si_003.pdf [file jz3c01922_si_003.pdf]

jz-2023-01922w.R1

Name: Peer Review Information for "Non-Equilibrium Magneto-Conductance as a Manifestation of Spin Filtering in Chiral Nanojunctions"

First Round of Reviewer Comments

Reviewer: 1

Comments to the Author

The manuscript utilize DFT non-equilibrium quantum transport calculations with relative advanced methods to show spin polarization under finite bias in a two terminal chiral system breaking spatial symmetries. Furthermore, when a suitably magnetized detector is introduced in the system, the net spin accumulation translates into a finite magneto-conductance.

The manuscript is well written aside from some of the legends of Figure 1.

There are however major questions that should be asked and the manuscript should be accepted only after addressing these questions.

The main question would relate to spin contamination that could result when using the unrestricted formalism.

To check that the authors should show that the spin polarization is flipped when flipping the enantiomers, or flipping the magnetic field and therefore the probe.

Without this it is hard to say if the results are real

Further, the authors don't describe what is the number of atoms and molecule they compute.

Lastly, most experiments on CISS are done at room temperature. Would would the effect of temperature on the suggested results.

Reviewer: 2

Comments to the Author

The authors present non-equilirbium calculations involving electron transport through chiral molecules. It is established that spins accumulate and forms a spin density when the current is flowing. This induced magnetization will affect the ultimate conductivity of the system, and this may be an explanation of the observed breaking of the Onsager reciprocity principle. Careful attention is payed to spatial symmetries and time revesal symmetry, and mild conditions for the presence of the spin accumulation effect is presented.

The work is an important contribution to the understanding of the CISS effect in the case of electron transport with magnetic leads. It can be published as is.

Author's Response to Peer Review Comments:

The response to Reviewer 1 is enclosed in the attached document.

We have adapted the bibliography according to the criteria in point 2. One website (with URL) has been kept, namely in Ref (55) since the corresponding code cannot be indicated otherwise.

RESPONSE TO REVIEWER 1

"The manuscript is well written aside from some of the legends of Figure 1."

We believe that the legends are readable with a reasonable zoom, and the meaning of the quantities should be clear from the caption and the basic definitions in the main text.

"The main question would relate to spin contamination that could result when using the unrestricted formalism."

Numerically, the spin contamination resulting from unrestricted DFT may indeed play a role in the displayed results but in all cases the impact on the S^2 expected values were below 0.5%, as calculated by Gaussian after the self-consistenct procedure, hence we would not expect it to have a significant impact. In any case, this effect would not play a role in the qualitative analysis since the spin components that are vanishing remain so.

"To check that the authors should show that the spin polarization is flipped when flipping the enantiomers, …

We performed a sistematic study of the behaviour of the spin polarization (in particular, its possible reversal upon enantiomeric substitution) in a previous study, see Ref 27. In any case, the conclusion (supported by both theory and numerical calculations) is that the spin polarization is guaranteed to reverse sign upon enantiomeric substitution only when the two resulting junctions as a whole (i.e., including the electrodes) are related by a mirror plane that does not permute the electrodes.

… or flipping the magnetic field and therefore the probe."

Figure 2 already displays the values of the spin polarization for opposite magnetizations $\pm M$, namely via the quantity $\Delta P(M) = P(M) + P(-M)$. As explained in the document, the polarization is reversed ($\Delta P(M) = 0$) when inverting the magnetization only when $P(M = 0) = 0$, in agreement with the symmetry analysis.

"Further, the authors don't describe what is the number of atoms and molecule they compute."

The atoms that are explicitly included in the calculations are those depicted in the corresponding figures. We have added the sentence: "The scattering regions in the calculations include exactly the atoms depicted in the corresponding figures". It was somewhat implicit in the Computational Methods sence: "In ANT the electrodes outside the scattering region (not depicted in the figures) are described by…". The geometry files with the position of these atoms were also included in the submission.

"Lastly, most experiments on CISS are done at room temperature. What would the effect of temperature on the suggested results."

The role of temperature is likely important in the quantitive results, as we had reckoned in the introduction: "Our quantitative results may nevertheless need to be complemented by inelastic effects such as electron-phonon interactions, which are currently deemed important to explain the large measured values and their scaling with temperature…". A qualitative study including vibrational modes or magnons would most likely be interesting, but we believe it to be out of the scope of the present work.
